# Ultrastructural viewpoints on the interaction events of *Scedosporium apiospermum* conidia with lung and macrophage cells

**DOI:** 10.1590/0074-02760180311

**Published:** 2018-10-08

**Authors:** Ana Carolina Aor, Thaís P Mello, Leandro S Sangenito, Beatriz B Fonseca, Sonia Rozental, Viviane F Lione, Venício F Veiga, Marta H Branquinha, André LS Santos

**Affiliations:** 1 Universidade Federal do Rio de Janeiro, Centro de Ciências da Saúde, Instituto de Microbiologia Paulo de Góes, Departamento de Microbiologia Geral, Laboratório de Estudos Avançados de Microrganismos Emergentes e Resistentes, Rio de Janeiro, RJ, Brasil; 2 Universidade Federal do Rio de Janeiro, Centro de Ciências da Saúde, Instituto de Biofísica Carlos Chagas Filho, Laboratório de Biologia Celular de Fungos, Rio de Janeiro, RJ, Brasil; 3 Universidade Federal do Rio de Janeiro, Centro de Ciências da Saúde, Faculdade de Farmácia, Laboratório de Bioensaios Farmacêuticos, Rio de Janeiro, RJ, Brasil; 4 Universidade Federal do Rio de Janeiro, Centro de Ciências da Saúde, Instituto de Microbiologia Paulo de Góes, Departamento de Microbiologia Geral, Setor de Microscopia Eletrônica, Rio de Janeiro, RJ, Brasil; 5 Universidade Federal do Rio de Janeiro, Instituto de Química, Programa de Pós-Graduação em Bioquímica, Rio de Janeiro, RJ, Brasil

**Keywords:** Scedosporium apiospermum, host cells, adhesion, invasion, lung cells, macrophages

## Abstract

**BACKGROUND:**

*Scedosporium apiospermum* is a ubiquitous, emerging and multidrug-resistant fungal pathogen with still rather unknown virulence mechanisms.

**OBJECTIVES/METHODS:**

The cellular basis of the *in vitro* interaction between fungi and host cells/tissues is the determinant factor for the development of a successful *in vivo* infection. Herein, we evaluated the interaction of *S. apiospermum* conidia with lung epithelial (A549), lung fibroblast (MRC-5) and RAW 264.7 macrophages by light and scanning/transmission electron microscopy.

**FINDINGS:**

After 4 h of fungi-host cell contact, the percentage of infected mammalian cells and the number of fungi per infected cell was measured by light microscopy, and the following association indexes were calculated for A549, MRC-5 and macrophage cells: 73.2 ± 25.9, 69.7 ± 22.5 and 59.7 ± 11.1, respectively. Both conidia and germinated conidia were regularly observed interacting with the evaluated cells, with a higher prevalence of non-germinated conidia. Interestingly, nests of germinated conidia were evidenced at the surface of lung cells by scanning electron microscopy. Some germination projections and hyphae were seen penetrating/evading the mammalian cells. Furthermore, internalised conidia were seen within vacuoles as visualised by transmission electron microscopy.

**MAIN CONCLUSIONS:**

The present study contributes to a better understanding of *S. apiospermum* pathogenesis by demonstrating the first steps of the infection process of this opportunistic fungus.

*Scedosporium apiospermum* is a saprophytic filamentous fungus that emerged as an opportunistic pathogen able to cause localised and disseminated infections in both immunocompromised and immunocompetent individuals.^1^ ^,^ ^2^ One of the most common pathological manifestations caused by *S. apiospermum* is known as eumycetoma, a superficial and localised cutaneous and subcutaneous infection normally caused by traumatic events, for example burns and skin injuries, which lead to the inoculation of conidial cells and/or mycelial fragments directly into host tissues.^1^ ^,^ ^3^ *S. apiospermum* can also cause invasive and disseminated infections such as pneumonia, fungal ball, paranasal sinusitis, allergic bronchopulmonary mycosis, corneal inflammation, endocarditis and manifestations in the central nervous system. These types of clinical complications are more frequently diagnosed in transplanted individuals and patients with haematological malignancies, advanced human immunodeficiency virus infection, diabetes mellitus and chronic pulmonary disease (e.g., cystic fibrosis patients), in which *S. apiospermum* can disseminate to virtually any anatomical site.^1^ ^,^ ^4^ ^,^ ^5^ However, the lungs are the preferred target to initiate colonisation when the route of contamination is through the inhalation of the conidia, which can then reach the pulmonary alveoli and interact with the resident alveolar cells.^6^ ^,^ ^7^

It is well known that the interaction process between microorganisms and host cells is a crucial step for successful tissue colonisation.^2^ ^,^ ^8^ To ensure that an infection is established, fungi exhibit distinct invasion strategies, such as conidial germination, which is a key event in the pathogenesis of filamentous fungi in order to cross tissue planes when these organisms invade host cells.^2^ ^,^ ^9^ ^,^ ^10^ In this line area, our group has demonstrated that conidial cells of *S. apiospermum*, *S. aurantiacum*, *S. minutisporum* and *Lomentospora prolificans* germinate *in vitro* under different growth conditions, initially forming a germ tube-like projection that grows in size forming long hyphae.^2^ ^,^ ^9^ ^,^ ^11^ ^,^ ^12^ ^,^ ^13^ ^,^ ^14^ The interaction among these hyphae gives rise to a structure resembling a mature biofilm, which could contribute to the increased ability of the fungus to disseminate and evade host immune attacks.^12^ ^,^ ^13^

Remarkably, very little research has focused on understanding the interaction process between host cells and fungi belonging to the *Scedosporium* genus.^2^ ^,^ ^12^ ^,^ ^15^ The study of this important step is fundamental for providing information required to uncover possible mechanisms involved in the pathogenicity of *S. apiospermum*. In this context, and due to the lack of information, we have decided to ultrastructurally study the ability of *S. apiospermum* conidia to interact with non-phagocytic (alveolar epithelial and lung fibroblast cells) and phagocytic (macrophages) cells.

## MATERIALS AND METHODS

*Microorganism and growth conditions* - *S. apiospermum* strain RKI07_0416 was kindly provided by Dr Bodo Wanke (Evandro Chagas Hospital, Oswaldo Cruz Institute, Rio de Janeiro, Brazil) and was identified by means of molecular approaches by Dr Kathrin Tintelnot (Robert Koch-Institut, Berlin, Germany).^16^ The sequencing of the *ITS* region revealed that this strain belongs to clade 4 (*S. apiospermum sensu stricto*) according to the taxonomy previously proposed by Gilgado and co-workers.^17^ The fungus was maintained in Sabouraud (2% glucose, 1% peptone, 0.5% yeast extract) liquid culture medium for seven days at room temperature with orbital shaking.^16^ Conidia were grown at room temperature on Petri dishes containing potato dextrose agar (PDA; Difco Laboratories, EUA). After seven days, conidial cells were obtained by washing the plate surface with phosphate-buffered saline (PBS; 10 mM NaH_2_PO_4_, 10 mM Na_2_HPO_4_, 150 mM NaCl, pH 7.2) and filtering through a 40-μm nylon cell strainer (BD Falcon, EUA) to remove hyphal fragments.^18^ The conidial cells were counted in a Neubauer chamber to adjust the cell ratio for the assays.

*Mammalian cell lineages and growth conditions* - A549 (ATCC CCL-185, alveolar basal epithelial cell of human adenocarcinoma), MRC-5 (ATCC CCL-171, fibroblast of human foetal lung) and RAW 264.7 (ATCC TIB-71, murine macrophages) cells were cultivated in 75-cm^2^ sterile culture bottles containing Dulbecco’s modified Eagle medium (DMEM) supplemented with 10% heat-inactivated foetal bovine serum (FBS) and 2 mM L-glutamine at 37ºC in an atmosphere of 5% CO_2_. For light and scanning electron microscopy (SEM) experiments, the mammalian cells (10^5^/well) were cultivated for 12 h over sterile glass coverslips placed into 24-well plates containing 0.5 mL DMEM with 10% FBS. For transmission electron microscopy (TEM), the mammalian cells (10^5^) were inoculated into 25-cm^2^ culture flasks containing 7 mL of DMEM supplemented with 10% FBS where they remained for 48 h.

*Fungi-mammalian cell association indexes* - Conidia (10^6^) of *S. apiospermum* were resuspended in fresh DMEM (without FBS) and allowed to interact with A549, MRC-5 and RAW macrophages (10^5^ cells/well) for 4 h, at 37ºC, 5% CO_2_. The conidia:mammalian cell ratio was adjusted to 10:1 by counting individually each cell type in a Neubauer chamber. After the interaction period, the wells were washed with DMEM to remove unattached conidia, fixed in Bowin’s solution and stained with Giemsa. The percentage of infected mammalian cells and the number of fungi per infected cell were determined by randomly counting 200 mammalian cells on each triplicate coverslip from three different experimental sets. Data were expressed as the mean ± standard deviation. The association index was also calculated by multiplying the mean number of fungi per infected mammalian cell by the percentage of infected cells. The images and counting were obtained using an Axioplan 2 light microscope (Jena Zeiss, Germany).^9^

*SEM* - For the observation of the structural aspects of the interaction, A549, MRC-5 and RAW cells were infected (conidia:mammalian cell ratio 10:1) and then processed for SEM analysis. Briefly, the systems were incubated at 37ºC for 4 h, washed three times with DMEM and fixed in 0.15 M sodium cacodylate buffer containing 2% paraformaldehyde and 2% glutaraldehyde, pH 7.4, for 16 h. After the fixation step, the samples were washed again three times with 0.15 M sodium cacodylate buffer, post-fixed for 90 min in 1% osmium tetroxide in 0.15 M sodium cacodylate buffer, washed one more time and dehydrated in an ascending ethanol series (50, 70, 80, 95 and 100%) for 10 min each. Next, the samples were dried using the critical point method, mounted on stubs, coated with colloidal gold and observed using a QUANTA 250 - FEI scanning electron microscope.^19^

*TEM* - To deeply image the ultrastructural aspects of the interaction process, A549, MRC-5 and RAW cells were infected with conidia (conidia: mammalian cell ratio 10:1) for 4 h. Then, the systems were washed with DMEM and fixed with 0.1 M cacodylate buffer containing 2.5% glutaraldehyde, 4% paraformaldehyde, 0.2% picric acid and 5 mM CaCl_2_. After 1 h of fixation, the cell monolayer was carefully removed from the bottles with the aid of a cell scraper and transferred to a vial. Subsequently, the cells were dehydrated in ethanol and embedded in resin (Unicryl) at -20ºC. Ultrathin sections of the resin were placed in nickel grids and incubated in 0.1 M Tris-buffered saline pH 7.5 (TBS; 20 mM Tris, 385 mM NaCl, 0.05% Tween 20) and 50 mM ammonium chloride. After three washes in TBS, staining with uranyl acetate and lead citrate was performed, and the ultrathin sections were observed using a transmission electron microscope (Zeiss 10C).

*Statistical analyses* - The microscopic images are representative images of three independent experiments performed in triplicate. When necessary, data were analysed statistically by means of one-way analysis of variance (ANOVA) using GraphPad Prism software 6.0 (GraphPad Software Inc., La Jolla, CA), in which *P* values ≤ 0.05 were considered statistically significant.

## RESULTS AND DISCUSSION

Some authors have reported that after inhalation of conidia, their adhesion to host cells/tissues, followed by germination and hyphal formation, seem collectively to be the main mechanisms of pathogenicity of infections caused by filamentous fungi.^10^ ^,^ ^20^ ^,^ ^21^ Thus, study of the interaction process between filamentous fungi and host cells is extremely important for a better understanding of this fundamental step. In *Aspergillus fumigatus,* the interaction events generate signals that induce host cells to surround and involve (until pathogen internalisation) approximately one-third of all conidia, while the non-internalised ones germinate externally and penetrate the cells, thus causing host cell damage.^6^ ^,^ ^8^ An *in vivo* model of allergic bronchopulmonary aspergillosis revealed that hyphae of *A. fumigatus* grow on and between the epithelial cells, suggesting that the development of hyphae in tissues is a subsequent event dependent on the initial binding of the conidia to the cell surface.^22^

**Fig. 1:**
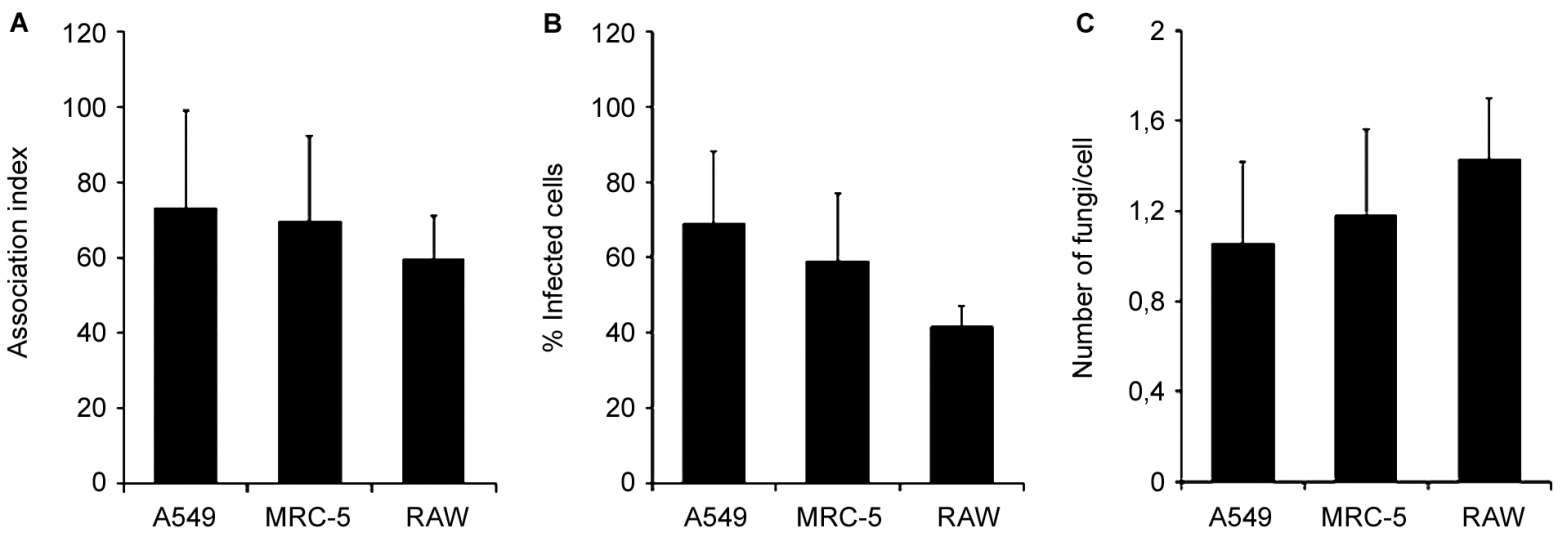
interaction of *Scedosporium apiospermum* with A549, MRC-5 and RAW mammalian cells for 4 h at 37ºC. The association indices of *S. apiospermum* with A549 alveolar pulmonary cells, MRC-5 fibroblast pulmonary cells and RAW murine macrophages (A); the percentage of infected A549, MRC-5 and RAW cells (B); and the mean number of fungi per infected cell (C) are shown. The data are expressed as the mean ± standard deviation (SD) of three independent experimental sets.

**Fig. 2:**
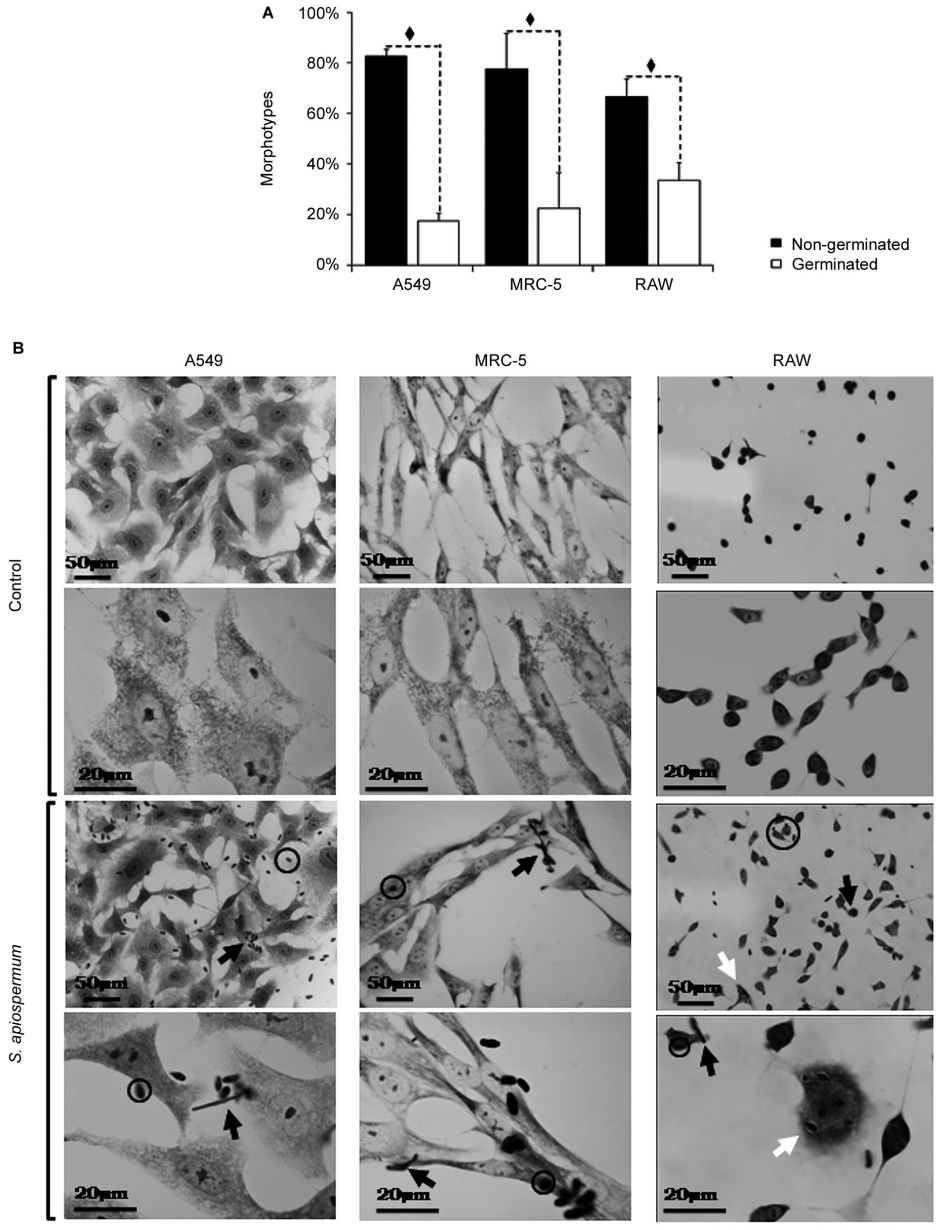
*Scedosporium apiospermum* morphotypes observed during the interaction process with A549, MRC-5 and RAW mammalian cells after 4 h at 37ºC. (A) Percentages of distinct morphotypes, germinated and non-germinated conidia interacting with A549, MRC-5 and RAW cells. The data are expressed as the mean ± standard deviation (SD) of three independent experimental sets. The diamonds in (A) represent the significant differences between the germinated and non-germinated conidia in each group (p < 0.05). (B) In the bright field microscopy, open circles demonstrate non-germinated conidia, white arrows show host cells with several internalised conidia, and black arrows point to germinated conidia. The mammalian cells submitted to the interaction process did not show visible morphological changes, with the exception of RAW cells, which became more sprawling after contact with the fungus. The micrographs are representative images of three independent experimental sets.

Due to the shortage of data concerning the interaction processes between host cells and that important opportunistic fungal pathogens belong to the *Scedosporium* genus, in the current work, we decided to study the co-cultivation of *S. apiospermum* conidia and some target cells. To this end, we chose MRC-5 (human foetal lung fibroblasts) and A549 (human lung epithelial cells) as target cell lineages, which represent the primary site of respiratory infections, as well as RAW macrophages as a representative phagocytic cell of the innate immune system. After only 4 h of contact, conidia were able to adhere to all three cell types and, as expected, to be internalised by RAW macrophages. The association indexes between the mammalian cells and *S. apiospermum* were 73.2 ± 25.9, 69.7 ± 22.5 and 59.7 ± 11.1 for A549, MRC5 and RAW, respectively. Herein, there was no significant difference among the association indexes of the fungus with the mammalian cells tested (Fig. 1A). The percentage of infected mammalian cells and the mean number of fungi per infected cell are also summarised in Fig. 1B and Fig. 1C, respectively. In addition to the observation of resting conidia, we could also see that a significant number of germinated conidia interacted with the mammalian cells. In this sense, germinated conidia represented 17.5 ± 2.8%, 22.6 ± 12.1% and 33.5 ± 7.1% of the total number of fungi that interacted with A549, MRC-5 and RAW cells, respectively (Fig. 2A). The number of germinated conidia that interacted with all of the mammalian cells tested was significantly lower when compared with the number of non-germinated conidia (Fig. 2A - diamonds). Corroborating this observation, light microscopy analyses demonstrated that conidia randomly attached to the mammalian cells, remaining as the predominant morphotype in relation to the germinated forms (Fig. 2B). After 24 h of interaction, all of the conidia differentiated into hyphae (data not shown), which covered the entire surfaces of the mammalian cells and rendered quantification impossible, as similarly reported by Mello and co-workers.^12^

**Fig. 3:**
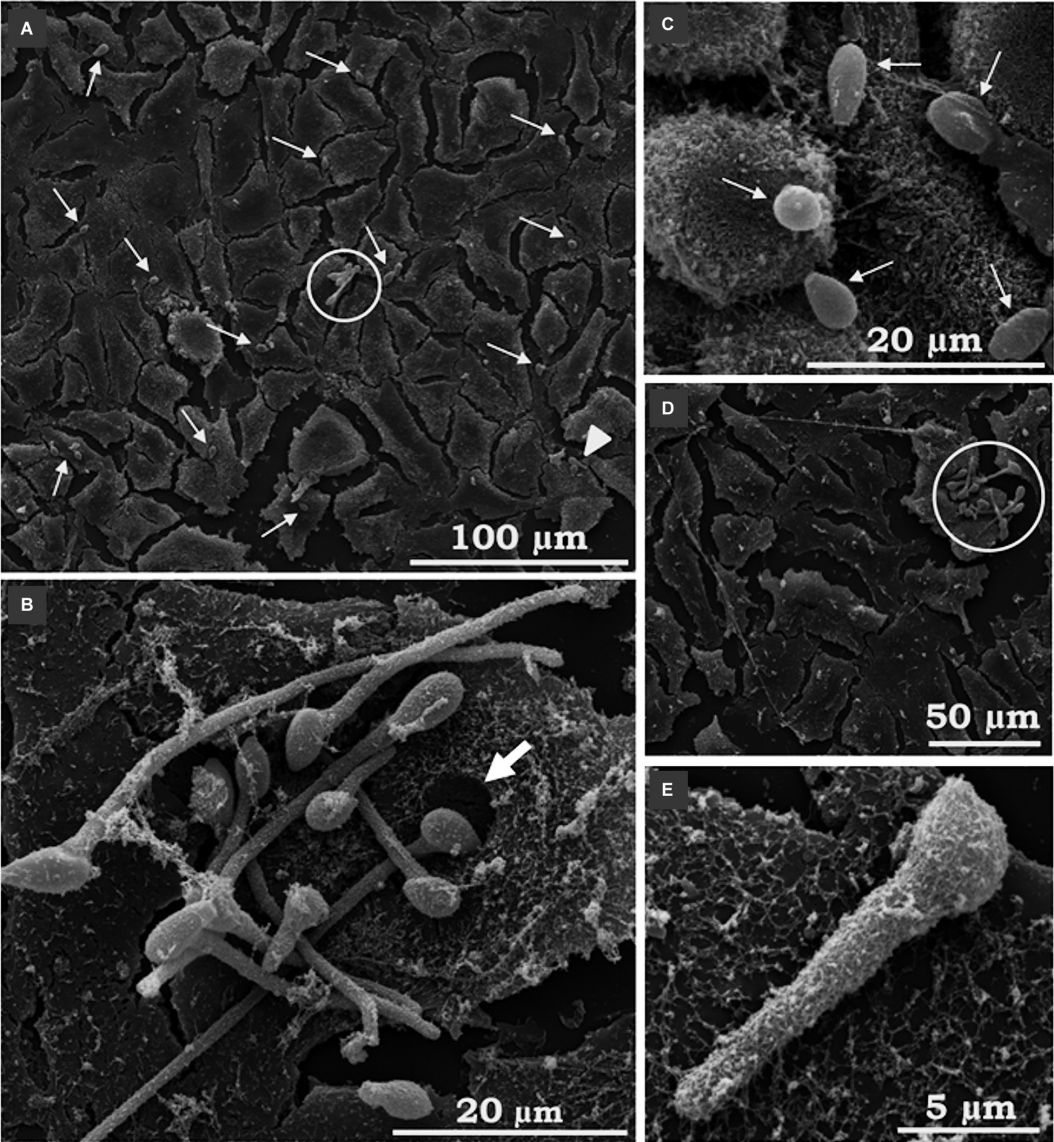
scanning electron microscopy (SEM) of the interaction between *Scedosporium apiospermum* conidia and A549 cells. The images revealed many conidial cells adhered to animal cells and some germinated conidia (A-E). Thin white arrows indicate the associated conidia; the white arrowhead shows dispersed germinated conidia, while the open circles show “nests” of germinated conidia. The thick white arrow points to a hollow corresponding to the exact location where the conidium was anchored to the mammalian cell (B). A filamentous network produced by epithelial cells can be seen surrounding germinated conidia (E). The micrographs are representative images of three independent experimental sets.

Previously, our group also described the ability of *S. apiospermum* to interact with HEp-2 laryngeal epithelial cells. Adherent conidia were observed after 1 h of incubation, and the association index reached a plateau between 2 and 4 h. After 2 h of interaction, conidia began to germinate, and after 4 h, they were already able to penetrate the membranes of the epithelial cells. It was also reported that the surface peptidorhamnomannan (PRM) molecules are recognised by host cell receptors, such as TLR4, characterising it as a classical *S. apiospermum* adhesin.^9^ The filamentous fungus *L. prolificans* (formerly *Scedosporium prolificans*) can interact with peritoneal macrophages, and its surface PRM also participates in this crucial event.^23^ Similarly, in *A. fumigatus*, it was reported that a small number of conidia were internalised and that most of them remained adhered to the external surface of the A549 cells. Those adhered conidia could externally germinate, generating long hyphae after 24 h of fungi-host cell contact.^24^ Similarly, *in vivo* experiments with *A. fumigatus* demonstrated that after 4 h of infection, conidia were in the beginning of the germination process and that after the 16 h time point, the formation of long hyphae was seen in the infected lungs of BALB/c mice. Lung sections from those mice revealed a strong pulmonary immune cell infiltration due to the hyphal growth.^25^

**Fig. 4:**
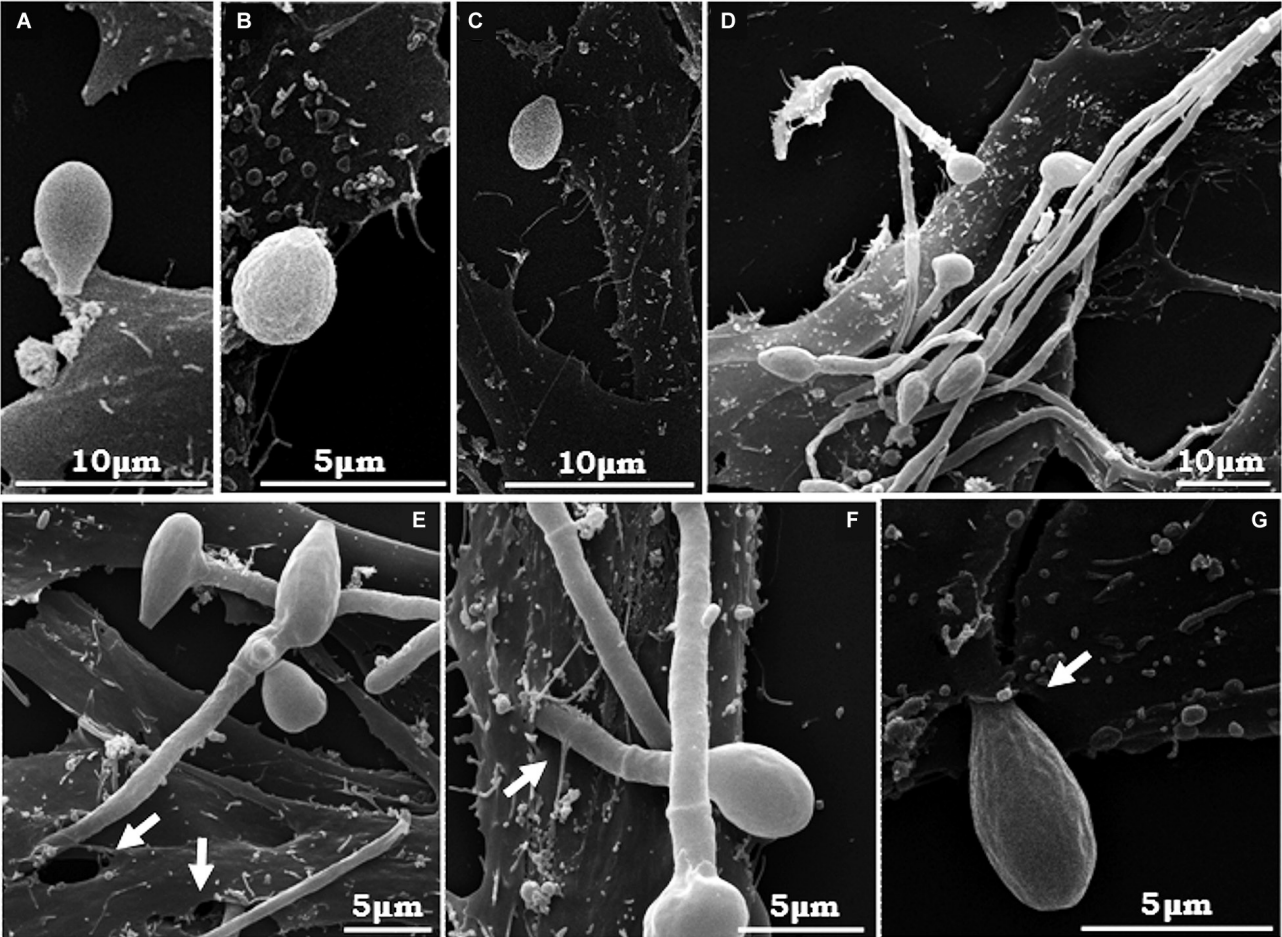
scanning electron microscopy (SEM) of the interaction between *Scedosporium apiospermum* conidia and MRC-5 fibroblasts. Fungal cells were placed in contact with the animal cells for 4 h. Conidial cells adhered to animal cells can be observed (A, B, C), as well as many grouped germinated conidia on the surface of the fibroblasts (D). In E, F and G, it is possible to see hyphae penetrating into the animal cells forming holes and cavities (white arrows) corresponding to the invasion sites. The micrographs are representative images of three independent experimental sets.

**Fig. 5:**
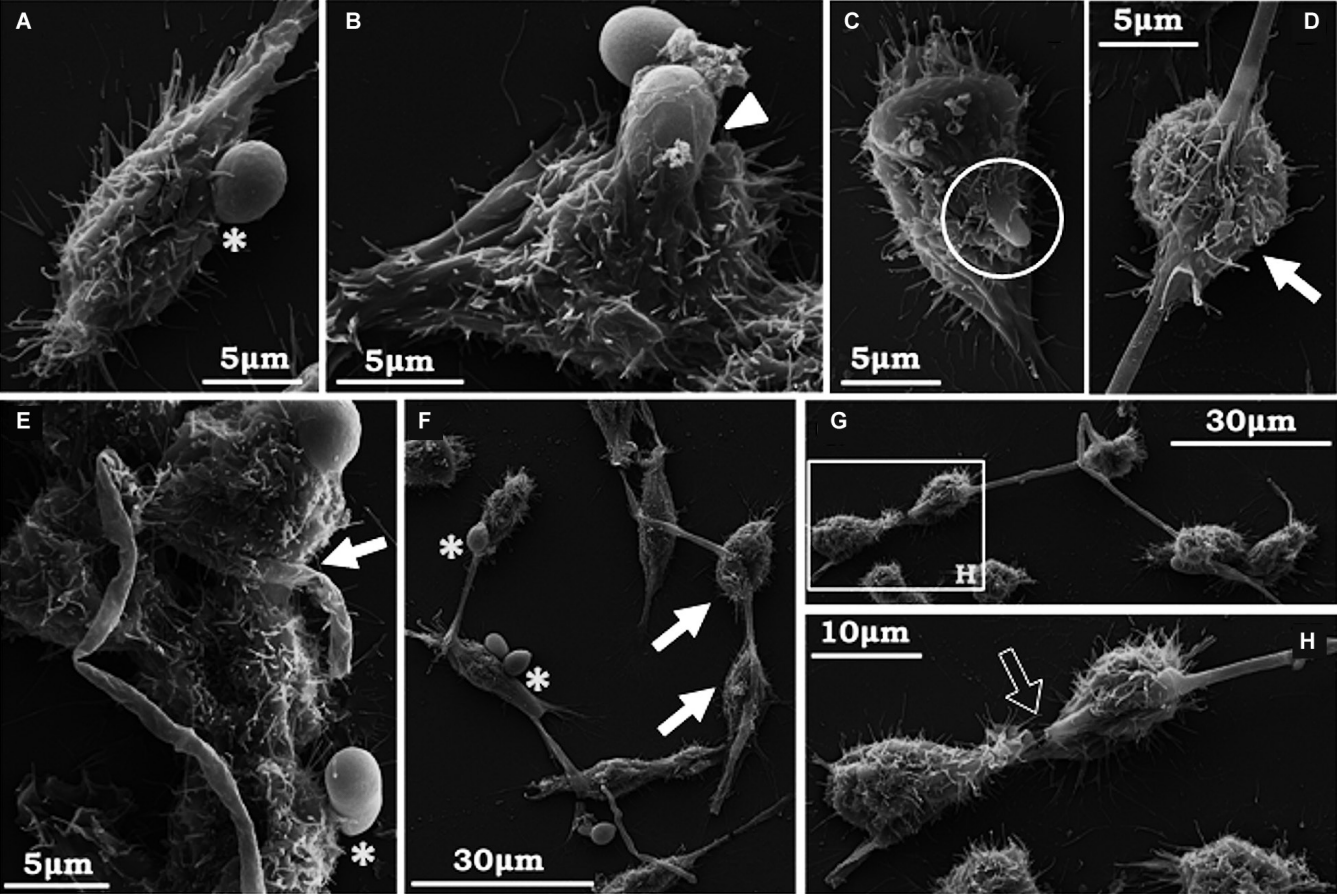
scanning electron microscopy (SEM) of the interaction between *Scedosporium apiospermum* conidia and RAW macrophages. Conidia, germinated conidia and hyphae appear simultaneously on and inside the phagocytes. RAW macrophages can be seen touching the conidia at an initial stage (asterisks - A, E, F) or at a later stage, where the conidia appear almost completely phagocytosed (arrowhead - B). A phagocytosed conidium can be seen breaking the macrophage cytoplasmic membrane and reaching the extracellular medium via germination growth (circle - C). The white arrows denote macrophages engulfing the fungal hyphae through extensions of their cytoplasm (D - G). E, F and G show more than one macrophage trying to hold the same long hyphae. The magnification in H shows the possible breaking of a hyphae caused by phagocytosis by two macrophages (open arrow). The micrographs are representative images of three independent experimental sets.

Corroborating the light microscopy, SEM images showed germinated and non-germinated conidia (with a predominance of the latter morphotype) adhered to A549 (Fig. 3A-E), MRC-5 (Fig. 4A-G) and RAW (Fig. 5A, B, F) cells, after 4 h of interaction. Despite the predominance of non-germinated conidia, we opted to highlight in our SEM images the germinated conidia and hyphae due to the further consequences of these morphotypes to the host cells. Although the pattern of conidia attachment was random, it was possible to observe several nests of germinated conidia over the A549 (Fig. 3B, D) and MRC5 (Fig. 4B) cells. No fungal nest was observed in RAW macrophages. The formation of nests or clusters was also demonstrated in *A. fumigatus* conidia over the surface of A549 cells.^20^ We hypothesised that the formation of fungal nests is one of the initial steps for building a complex and robust biofilm structure, which is preceded by the adhesion to a substrate and the germination of the conidia into hyphae.^2^ Corroborating this statement, our group previously reported for the first time the ability of *S. apiospermum* to form a cohesive, multicellular and robust structure that resembles a biofilm, which contains a dense network of intertwined hyphal cells covered with extracellular matrix on both abiotic (polystyrene, glass, catheters made from polyvinyl chloride, siliconised latex and polyurethane) and biotic (A549 cells) surfaces.^12^ ^,^ ^13^

In particular, A549 cells apparently secreted a fibrous-like network that appeared to surround the adhered conidia and germinated conidia of *S. apiospermum* (Fig. 3B, E). This phenomenon is presumably due to the particular secretory properties of the A549 pulmonary cells. A549 cells possess numerous surface villus-like projections and contain lamellar bodies similar to type II pneumocytes. These various electron-dense lamellar bodies are responsible for the vesicular aspect of the cytoplasm when viewed by optical microscopy.^26^ In our study, the fibrous-like substance was not seen in MRC-5 and RAW images; however, a similar substance to this network was reported in the interaction between *Candida albicans* and corneocyte cells. *C. albicans* blastoconidia were coated with strands or fibrils of amorphous material on the epidermal corneocyte surface. This material had an appearance of stretch strands, resembling a bridge between the blastoconidia and the corneocytes. Similar to our results, this “film-like” substance was noted covering the cells near the sites of blastoconidia attachment.^27^ The authors suggested that this substance is similar to an extracellular mucus-like material that has been previously noted in SEM studies of *Candida* adherence to gastrointestinal epithelium^28^ and vaginal epithelial cells.^29^

It should be pointed out that, in general, the morphology and density of the monolayers of all three of the mammalian cell lineages studied herein were preserved at the end of 4 h of interaction with *S. apiospermum* (Figs 2B, 3, 4, 5). However, RAW macrophages became more sprawling after contact with the fungus, probably due to cell activation and phagocytic mechanisms (Figs 2B, 5). Furthermore, only punctual damages were visualised at the fungal anchor sites during the interaction process (Figs 3, 4, 5). In the interaction between A549 cells and *S. apiospermum*, *S. minutisporum* or *S. aurantiacum*, little damage was observed after 24 h of fungus contact, while *L. prolificans* completely destroyed the epithelial cell monolayer at this time point.^12^ In *A. fumigatus*, even with the formation of hyphae at 12 h of incubation, detachment of cells from the culture plates was only clear after 24 h of interaction.^20^ In another work, the authors also reported that despite the presence of many adhered germinated conidia in the first hours, *A. fumigatus* did not cause morphological damage in A549 cells until complete mycelium formation (24-48 h), when monolayer detachment was seen.^24^

In our results, conidia (Fig. 3B), projections of germinated conidia and hyphae of *S. apiospermum* (Fig. 4C-E) were endocytosed by the A549 and MRC-5 cells. Interestingly, it was possible to visualise holes corresponding to the respective endocytosis sites (Figs 3B, 4C-E). Alternatively, the possibility that endocytosed conidia may have germinated within the host cell cannot be ruled out. Using RAW cells, conidia, germinated conidia and hyphae can be seen simultaneously on the phagocytes and have been internalised by them. Even if phagocytosed, the *S. apiospermum* conidia can germinate within the macrophages, forming germ tube-like projections that can lyse the membrane at this point to reach the extracellular medium, which is a remarkable aspect of this interaction (Fig. 5C). However, this event may also be the result of the endocytosis of previously germinated conidia. In addition, RAW macrophages can be seen (i) touching the conidia via pseudopod formation (Fig. 5A, E, F); (ii) trying to engulf long hyphae alone or together with neighbouring macrophages (Fig. 5D-H); and (iii) completing the engulfment process of conidia (Fig. 5B). The cavities and holes observed in the regions of contact with the conidia and on the invasion sites of germinated conidia suggest an active cavitation process affecting the host cell surface. Similar results were found in the interplay between *Cryptococcus neoformans* and A549 cells and *Paracoccidioides brasiliensis* and Vero cells. In those studies, the depressions were observed in the exact spots where the yeast was anchored to the cell.^30^ ^,^ ^31^ In *A. fumigatus*, several conidia were observed in association with non-ciliated bronchial epithelial cells with indentations on the surface, resembling craters, after 6 h of incubation,^32^ while in our work, the indentations on the surface of the host cells could be observed earlier, even after 4 h. The holes at the invasion sites of germinated conidia were also reported for *C. albicans* hyphae. Depressions on epithelial cell surfaces caused by hyphae invasion were previously demonstrated, indicating active penetration.^33^ Invasion of cells via active penetration presumably relies on a combination of physical pressures exerted by the extending hypha and on the secretion of hydrolytic enzymes. However, the exact mechanism underlying active penetration and other factors involved in this process remain unknown.^18^ It has already been described in *S. apiospermum* that the penetration into host cells can be partially due to the secretion of hydrolytic enzymes. Indeed, two extracellular metallopeptidases from *S. apiospermum* were able to cleave laminin, fibronectin (extracellular matrix glycoproteins present in lung tissue) and in degrading mucin (*O*-linked glycoproteins produced and secreted by the epithelial mucosa). The degradation of these glycoproteins could help invasive fungus to migrate into adjacent tissues or to reach the bloodstream.^16^

**Fig. 6:**
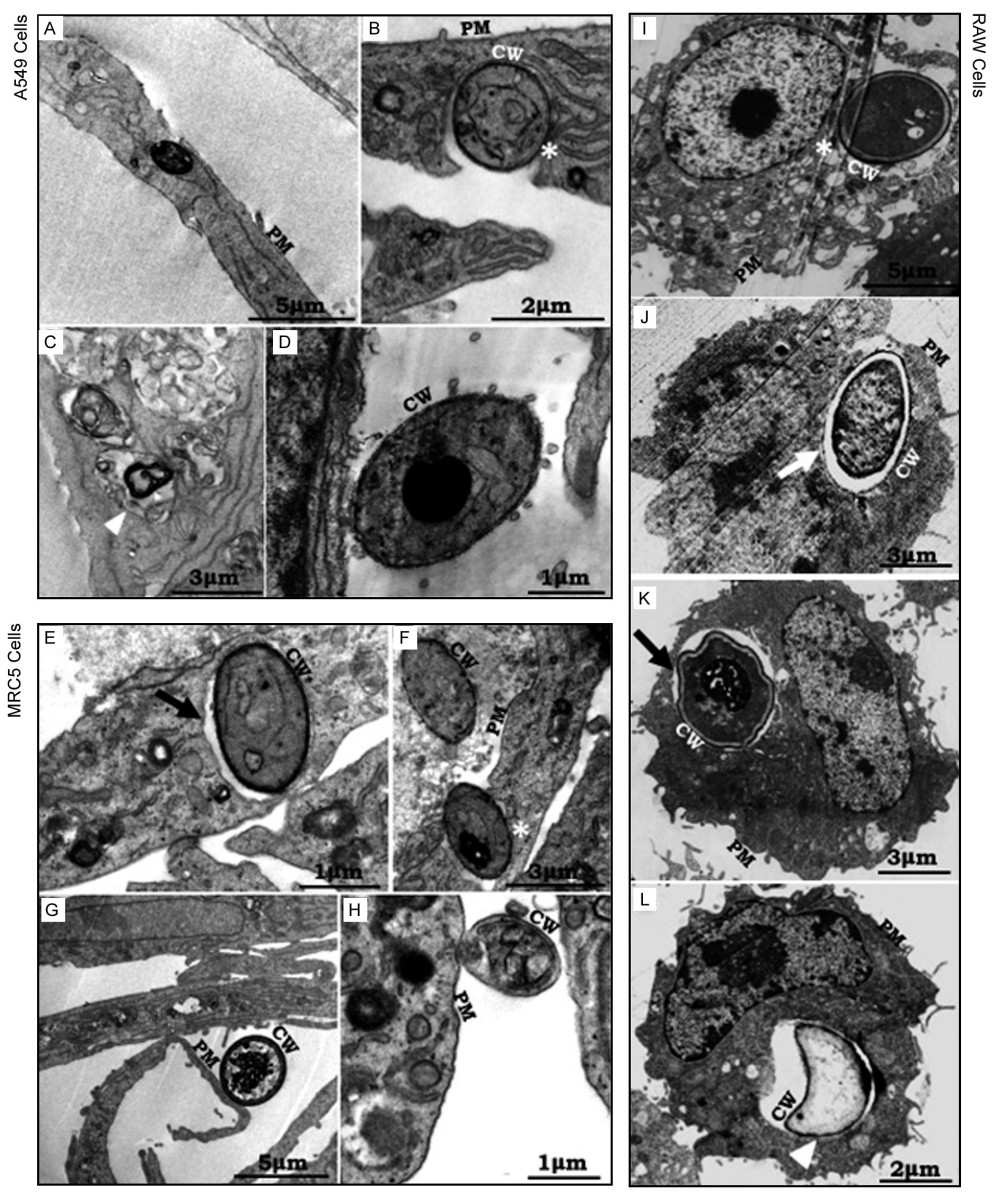
transmission electron microscopy (TEM) of the interaction between *Scedosporium apiospermum* conidia and A549 epithelial cells (A-D), MRC-5 fibroblasts (E-H) and RAW (I-L) macrophages. Internalised conidia can be viewed in the absence (A, F) or in the presence of a vacuole (E, J, K). The vacuole membrane can be visualised (white arrow). Black arrows show fungi inside the vacuole. B, F and I show the exact moment that conidia invade the animal cell (white asterisks). In C and L, a conidial cell is seen inside a late endosome, already in the degradation process (white arrowhead). Note that in D, G and H the presence of adhered fungi to the animal cells. PM indicates the location of the plasma membrane of the animal cells, whereas CW shows the fungal cell wall. The micrographs are representative images of three independent experimental sets.

For a deeper view of our interaction systems, complementary analyses were performed by TEM. The images demonstrated that conidia of *S. apiospermum* adhered to and were endocytosed by epithelial cells (Fig. 6A-D), fibroblasts (Fig. 6E-H) and phagocytosed by macrophages (Fig. 6I-L) after 4 h of incubation. Through these experiments, we could also confirm that the exclusive adhesion of conidia is a recurrent event during the interaction. Likewise, adhered conidia are the primary occurrence (more than 90%) in the interaction between *A. fumigatus* and A549 cells.^20^ In addition, we could see cytoplasmatic membranous formations around and engulfing the conidia (Fig. 6B, F, I) and some internalised conidia inside vacuoles (Fig. 5E, J, K). Representative images of late endosomes with internal material already in the degradation process can also be observed inside A549 (Fig. 6C) and RAW (Fig. 6L) cells. The presence of vacuoles suggests the presence of endocytosis, which occurs through the formation of pseudopod-like structures by the host cell.^19^ ^,^ ^33^ ^,^ ^34^ Conidia internalised in vacuoles were also visualised by TEM after 4 h of interaction between *A. fumigatus* and human nasal epithelial cells.^35^ In other works with *P. brasiliensis*, some fungi were observed inside vacuoles of both Vero and HeLa cells.^19^ ^,^ ^36^

Interestingly, a study with *C. albicans* suggested that endocytosis occurs early during epithelial cell-*C. albicans* interaction - usually within 4 h.^37^ This kind of endocytosis (called induced or mediated) seems to be a host cell-driven process that involves significant changes in the cytoskeleton conformation of the host cells and the fungus.^34^ ^,^ ^36^ These changes suggest that a permissiveness of the fungal cells to be internalised may be important in the development of disease, even though the fungus is not an obligate intracellular pathogen. In an interesting study, Amitani and Kawanami^32^ inoculated *A. fumigatus* into organ tissue cultures. The results suggested that the fungi could invade host cells at least by penetration of the hyphae through the intercellular spaces in the epithelium, by direct penetration of hyphae through epithelial cells or by internalisation of conidia. Phagocytes are especially important effector cells in the control (or attempted control) of systemic mycoses, and serve a fundamental role in innate immunity through internalisation of the fungus.^24^ ^,^ ^38^ In this context, the presence of a-glucans on the *S. apiospermum* conidial surface was demonstrated to be important for phagocytosis by peritoneal macrophages and for the activation of these cells.^39^

*In conclusion* - Collectively, our study suggests that after the initial binding of *S. apiospermum* conidia to host cells, they are internalised. In parallel, some extracellular conidia quickly trigger a differentiation programme, which leads to the formation of a germ tube-like projection. In short, it was possible to see two invasion mechanisms: intracellular conidia, suggesting endocytic uptake, and direct hyphal penetration. Although induced endocytosis and active penetration can be considered distinct mechanisms, these processes play complementary roles during the invasion process and the establishment of an infection.^18^ Together, our results contribute to the knowledge on the study of the association process between *S. apiospermum* and its host cells and to a better understanding of the cellular mechanisms involved in the first steps of the pathogenesis in scedosporiosis.
